# Indication adherence and outcome of post-operative radiotherapy in oral cavity cancer patients with intermediate adverse pathological tumor features: A nationwide population-based analysis

**DOI:** 10.1016/j.ctro.2026.101157

**Published:** 2026-04-04

**Authors:** Hanneke Doremiek van Oorschot, Jose Angelito Hardillo, Frank J.P. Hoebers, Joris B.W. Elbers, Robert Jan Baatenburg de Jong, Dutch head and neck audit - oral cavity collaborator group, Robert J.J. van Es, Guido B. van den Broek, Robert Paul Takes, Gyorgy Bela Halmos, Dominique Valerie Clarence de Jel, Richard Dirven, Martin Lacko, Lauretta Anna Alexandra Vaassen, Jan-Jaap Hendrickx, Marjolijn Abigal Eva-Maria Oomens, Hossein Ghaeminia, Jeroen C. Jansen, Annemarie Vesseur, Rolf Bun, Leonora Q. Schwandt, Christiaan A. Krabbe, Thomas J.W. Klein Nulent, Alexander Jan Marcelis van Bemmel, Reinoud J. Klijn

**Affiliations:** e Department of Head and Neck Surgical Oncology, UMC Utrecht Cancer Centre, Utrecht, the Netherlands; f Department of Otorhinolaryngology, Head and Neck Surgery, Radboud University Medical Center, Nijmegen, the Netherlands; g Department of Otorhinolaryngology/Head and Neck Surgery, University Medical Centre Groningen, University of Groningen, Groningen, the Netherlands; h Department of Head and Neck Oncology and Surgery, Netherlands Cancer Institute/Antoni van Leeuwenhoek, Amsterdam, the Netherlands; i Department of Otorhinolaryngology and Head and Neck Surgery, GROW School for Oncology and Reproduction, Maastricht University Medical Center, Maastricht, the Netherlands; j Department of Cranio-maxillofacial surgery, GROW School for Oncology and Reproduction, Maastricht University Medical Center, Maastricht, the Netherlands; k Department of Otorhinolaryngology, Head and Neck Surgery, Amsterdam UMC Location VUMC, Amsterdam, the Netherlands; l Cancer Center Amsterdam, Amsterdam, the Netherlands; m Department of Oral and Maxillofacial Surgery, Rijnstate Hospital, Arnhem, the Netherlands; n Department of Otorhinolaryngology, Head and Neck Surgery, Leiden University Medical Centre, Leiden, the Netherlands; o Department of Otolaryngology, Head and Neck Surgery, Elisabeth Tweesteden Ziekenhuis Tilburg, Tilburg, the Netherlands; p Department of Oral and Maxillofacial Surgery/Head and Neck Oncology Noordwest Hospital group, Alkmaar en Dijklander Hospital, Hoorn, the Netherlands; q Department of Oral and Maxillofacial Surgery, Medical Centre Leeuwarden, Leeuwarden, the Netherlands; r Department of Oral and Maxillofacial Surgery, Haaglanden Medical Center, The Hague, the Netherlands; s Department of Otorhinolaryngology and Head and Neck Surgery, Medisch Spectrum Twente, Enschede, the Netherlands; t Department of Oral and Maxillofacial and Head and Neck Surgery, Medisch Spectrum Twente, Enschede, the Netherlands; a Department of Otorhinolaryngology and Head and Neck Surgery, Erasmus Medical Centre Cancer Institute, Rotterdam, the Netherlands; b Scientific Bureau, Dutch Institute for Clinical Auditing, Leiden, the Netherlands; c Department of Radiation Oncology (MAASTRO clinic), GROW School for Oncology and Developmental Biology, Maastricht University Medical Centre+, Maastricht, the Netherlands; d Department of Radiotherapy, Erasmus Medical Centre Cancer Institute, Rotterdam, the Netherlands

**Keywords:** Head and neck cancer, Oral Cavity Cancer, Postoperative radiotherapy, Real-world data

## Abstract

**Purpose:**

The significance of intermediate-risk adverse pathological features in oral squamous cell carcinoma (OSCC) remains unclear. Consequently, the indications for postoperative radiotherapy (PORT) vary between hospitals. This study evaluates treatment patterns and outcomes of PORT for OSCC patients with intermediate-risk adverse pathological features in a real-world national cohort.

**Materials and methods:**

All first primary OSCC patients (n = 683) with intermediate-risk adverse pathological features from the tumor treated between 2018 and 2021 in the Netherlands were included. Features were 1–5 mm resection margins, pT3-T4 classification, perineural invasion, worst pattern of invasion (WPOI) 4–5, or vaso-invasive growth. Odds ratios (ORs) for receiving PORT were calculated using multivariable regression analysis. Hospital variation was assessed using funnel plots. Two-year overall survival (OS) and local control (LC) were evaluated using multivariable Cox regression analysis.

**Results:**

The percentage of patients receiving PORT increased when more features were present (4.0% for 1 feature, 67% for all features). Significant predictors for PORT were pT3-T4 (OR 23.3), 1–5 mm margin (OR 3.72), WPOI 4–5 (OR 1.83) and perineural invasion (OR4.75). Patients with vaso-invasive growth (OR 0.19) and increasing age (OR 0.96) received PORT less often. Hospital differences in PORT treatment remained after correction for confounders. PORT did not significantly improve the adjusted hazard ratio for LC (1.56, p = 0.36) and OS (0.55, p = 0.08).

**Conclusion:**

Of the tumor-related pathological features, pT3-pT4 contributed the most to PORT treatment in OSCC. Significant hospital differences remained after correction for confounding factors. Future research should focus on guideline consensus to prevent over- and undertreatment.

## Highlights

• Postoperative radiotherapy (PORT) can be indicated after oral cancer surgery.
• Due to guideline ambiguity, PORT indication based on tumor characteristics varies.
• This study observed significant hospital differences in local PORT indications.
• PORT did not significantly impact two-year local control and overall survival.
• Future research on guideline consensus could prevent over- and undertreatment.

## Introduction

Surgical resection is standard treatment for oral squamous cell carcinoma (OSCC).[1] When adverse pathological features are identified upon pathological evaluation of the resected specimen, postoperative radiotherapy (PORT) can be indicated to improve locoregional control and survival outcomes.[1] Chemoradiation is usually applied in case surgical margins < 1 mm as this is considered a high-risk adverse pathological feature. Debate remains on whether to treat patients with PORT in the presence of one or multiple intermediate-risk adverse pathological features from the tumor.[1], [2] Therefore the National Comprehensive Cancer Network (NCCN) and European Society For Medical Oncology (ESMO) recommend to consider PORT in the presence of intermediate-risk features. These features include close margins (1–5 mm), pT3 or pT4, perineural invasion, worst pattern of invasion (WPOI) 4–5, and vaso-invasive growth.[1], [3] Randomized trials on clinical outcome after OSCC surgery with PORT versus surgery alone are lacking. Review articles from retrospective small cohort studies fail to provide definitive advice on the PORT indications based on the tumor profile.[4], [5], [6].

Because the relative importance of intermediate-risk adverse tumor-related pathological features remains ambiguous, variability exists among different clinicians and centers in their recommendations for PORT (Dutch Head and Neck Society [NWHHT] survey 2024, unpublished). In the Netherlands, all OSCC patients are treated in specialized head and neck cancer hospitals and discussed pre- and postoperatively by the local multidisciplinary tumor board. The indication for local PORT is considered in light of the tumor-related adverse features and the patient's condition.[1], [7] The multidisciplinary team's advice is then discussed with the patient to make a final decision. However, deciding between possible worse oncological outcomes or radiotherapy-related toxicity poses a complex dilemma.

This study evaluates the contribution of intermediate-risk adverse pathological features to receiving local PORT for OSCC patients and between-hospital differences. Moreover, two-year follow-up outcomes of local PORT are assessed in a real-world national cohort.

## Methods

### Study design and data source

Data for this population-based cohort study were derived from the Dutch oral cavity cancer cohort.[8] This is a subset from the Dutch Head and Neck Audit (DHNA) database including variables on the treatment and follow-up of all first primary head and neck tumors.[9] Since 2018, all fourteen head and neck cancer centers in the Netherlands have contributed to the DHNA. The DHNA is one of 26 clinical quality registries maintained by the Dutch Institute for Clinical Auditing, where data standardization and verification are ensured.[10], [11] After reviewing this research proposal (MEC-2022–0816), the institutional research review board of the Erasmus Medical Center (Rotterdam, The Netherlands) granted it exemption from the Medical Research Involving Human Subjects Act.

### Cohort selection

All patients who underwent surgery with curative intent for first primary pT1-T4 OSCC between 2018 and 2021 were reviewed (ICD-O-3 codes C00, C02-C04, C05.0, C5.8–9, and C06.0–8).[12] Patients with positive margins or pN2a or higher on pathological assessment were excluded because these are high-risk pathological features with a clear indication for local and/or regional PORT.[7] The DHNA data does not distinguish between local, regional or locoregional PORT. Therefore, all patients with positive lymph nodes on pathology were excluded to prevent cohort contamination with patients that only received regional PORT. In addition, patients who underwent re-resection and those who underwent adjuvant chemoradiotherapy were excluded. Patients with one or more intermediate-risk adverse pathological tumor features were selected. Patients with missing variables on the intermediate-risk adverse pathological tumor features were excluded from analysis.

### Outcomes and definitions

The primary outcomes were patient and tumor characteristics associated with receiving PORT. Included patient and tumor characteristics were sex, age, World Health Organization (WHO) performance score, American Society of Anesthesiologists (ASA) score, pTN-classification following the 8th edition,[13] subsite oral cavity (tongue, gum, floor of mouth, other) based on the ICD-O-3 coding,[12] definitive margin, WPOI 4–5, perineural invasion, and vaso-invasive growth. Margins were considered positive when < 1 mm, close when 1–5 mm, and clear when > 5 mm following the Royal College of Pathologists guidelines.[14] The pNx-group includes cN0 patients that did not undergo a sentinel node procedure or neck dissection based on preoperative diagnostic and treatment protocols. Because data on a comorbidity score was missing in 44% of the patients and not at random (different registration adherence between hospitals), this variable was not included for analysis.

For two-year follow-up the local control (LC) and overall survival (OS) were selected as outcomes. The LC was defined as the duration from surgery to first detection of tumor at the primary site. Patients who passed away with metastasis or before any documented tumor progression were censored at the date of death. Patients who were alive after two years without signs of local failure were censored at the date of last follow-up. The OS was defined as the time from surgery to death from any cause. There were no patients with locoregional tumor recurrence before the end of radiotherapy.

### Statistical analysis

Statistical analysis was performed in R studio (version 4.2). Based on the distribution, continuous variables were displayed as a median with an inter-quartile range IQR) and categorical variables as proportions. P-values < 0.05 were considered as statistically significant. Missing data was imputed using multiple imputation (mice package) and for survival data the Nelson Aalen method was adhered.[15], [16] A propensity-score matched evaluation of survival outcomes was considered. This analysis was not performed because after matching the propensity scores of the treated (PORT) and untreated group were not evenly distributed (Appendix A).

The association between patient and tumor characteristics and receiving PORT was assessed using multivariable regression analysis and reported as odds ratios (OR) with confidence intervals (CI). Covariates were included based on the available degrees of freedom for all regression analyses. When not all characteristics could be included in the model, variables were selected using backwards selection. To evaluate the proportional contribution of each patient and tumor characteristic for receiving PORT, the Wald statistics minus the degrees of freedom (χ2 – df) was calculated and visualized.

Hospital variation in PORT treatment was visualized using funnel plots.[17] The uncorrected percentage of patients receiving PORT per hospital was calculated and compared to the benchmark, which is the national weighted average. To adjust hospital results for patient and tumor characteristics, a multivariable regression model was used to calculate the expected percentage of patients that would be treated with PORT per hospital. Then, the observed-expected ratio per hospital was calculated by dividing the actual percentage of patients treated with PORT by the expected percentage. An observed expected ratio > 1 means that the hospital treated more patients with PORT than was expected based on the estimated predicted probability in the national cohort.

Patients who had missing data follow-up data or had the event of interest (first detection of tumor progression or death of any cause) within six weeks after surgery were excluded from time to event analysis to prevent immortal time bias. Kaplan Meier curves for two-year LC and OS were presented to visualize two-year outcomes and stratified for receiving PORT including the log-rank test. Cox regression analysis was performed to calculate adjusted hazard ratio’s (aHR) for PORT.

## Results

### Study population

**Fig. 1:**
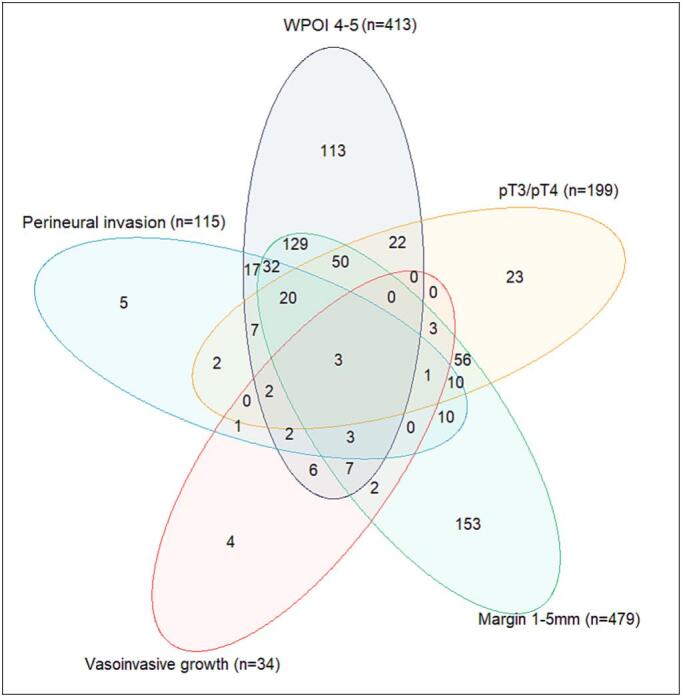
Caption: Venn diagram for adverse pathological features (n = 683). WPOI = worst pattern of invasion.

A total of 683 OSCC patients with intermediate-risk adverse pathological tumor features who received surgery between 2018 and 2021 were selected from the DHNA database (Appendix B). PORT was received by 148 patients (Table 1). When comparing patients who received PORT to surgery-only patients, PORT patients were younger (median 66 versus 68 years respectively, p = 0.009) and more often had a higher pT-classification (45% versus 7.9% T4 respectively, p=<0.001). From all adverse pathological features, 1–5 mm margins were observed the most (n = 479), followed by WPOI 4–5 (n = 413), pT3-T4 (n = 199), perineural invasion (n = 115), and vaso-invasive growth (n = 34, Fig. 1).

**Table 1:** Patient and tumor characteristics.

	PORT		
	No	Yes	
Characteristic	n = 535	n = 148	p-value1
Sex			0.078
Male	278 (52%)	89 (60%)	
Female	257 (48%)	59 (40%)	
Age (years, IQR)	68 (59, 78)	66 (59, 71)	**0.009**
WHO performance score			0.2
Normal activity (0)	267 (50%)	78 (53%)	
Symptomatic, fully ambulatory (1)	103 (19%)	24 (16%)	
Ambulatory > 50% of the time (2)	40 (7.5%)	10 (6.8%)	
Ambulatory < 50% of the time (3)	15 (2.8%)	0 (0%)	
Bedridden (4)	0 (0%)	0 (0%)	
Unknown	110 (21%)	36 (24%)	
ASA score			0.11
ASA I	39 (7.3%)	9 (6.1%)	
ASA II	283 (53%)	82 (55%)	
ASA III	163 (30%)	49 (33%)	
ASA IV	7 (1.3%)	4 (2.7%)	
Unknown	43 (8.0%)	4 (2.7%)	
Subsite oral cavity (ICD-O-3)			**<0.001**
Tongue	278 (52%)	42 (28%)	
Gum	70 (13%)	51 (34%)	
Floor of mouth	103 (19%)	25 (17%)	
Other subsites	84 (16%)	30 (20%)	
pT-classification			**<0.001**
pT1	254 (47%)	9 (6.1%)	
pT2	195 (36%)	26 (18%)	
pT3	44 (8.2%)	46 (31%)	
pT4	42 (7.9%)	67 (45%)	
pN-classification			**<0.001**
pNx	135 (25%)	10 (6.8%)	
pN0	400 (75%)	138 (93%)	
Definitive surgical margin			**<0.001**
Margin 1–5 mm	355 (66%)	124 (84%)	
Margin > 5 mm	180 (34%)	24 (16%)	
Perineural invasion	67 (13%)	48 (32%)	**<0.001**
WPOI 4–5	322 (60%)	91 (61%)	0.8
Vasoinvasive growth	30 (5.6%)	4 (2.7%)	0.2
Median follow-up time (days, IQR)	768 (174, 887)	810 (213, 881)	0.8
Unknown	9 (1.7%)	0 (0%)	

Abbreviations: ASA = American Society of Anesthesiologists, IQR = inter-quartile range, PORT = post-operative radiotherapy WHO = World Health Organization.

^a^p-values were calculated using the Pearson’s Chi-squared test, Wilcoxon rank sum test, or Fisher’s exact test where. Bold p-values are significant.

### Variables associated with receiving PORT

**Fig. 2:**
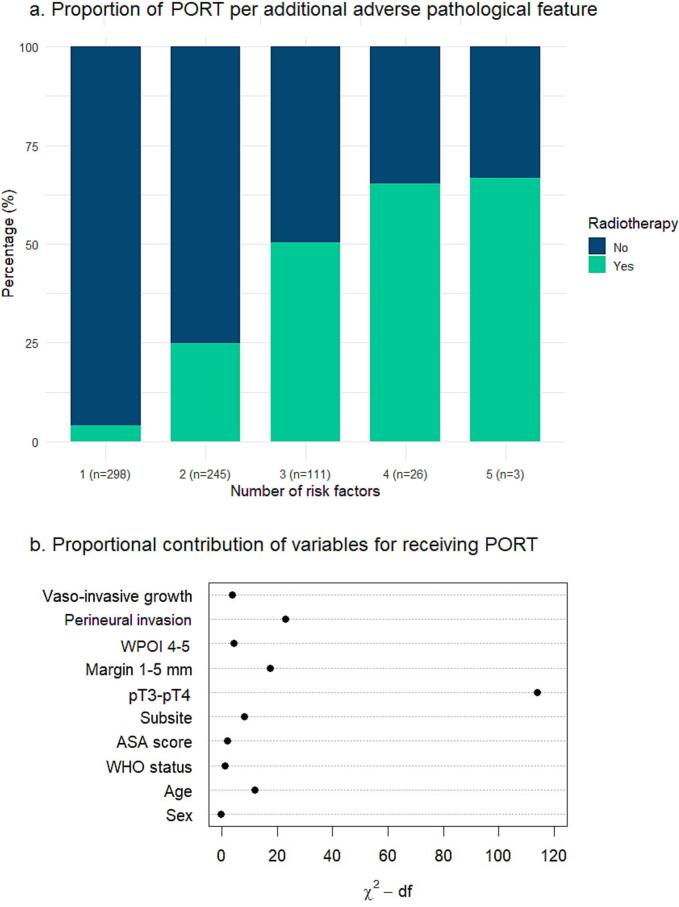
Variables associated with receiving post-operative radiotherapy (n = 683). Abbreviations: PORT = post-operative radiotherapy, WPOI = worst pattern of invasion.

The proportion of patients receiving PORT increased when more intermediate-risk adverse pathological features were present (Fig. 2a). When one feature was present (298/683 patients), 4.0% (n = 12) of the patients were treated with PORT. In case of two (245/683 patients) and three features (111/683 patients), 25% (n = 61) and 50% (n = 56) received PORT, respectively. Sixty-five percent (n = 17) of the patients with four features (26/683 patients) were treated with PORT. All five features were present in 3/682 patients and 2 of them (67%) received PORT.

Due to sufficient events, all patient and tumor characteristics could be included in the multivariable regression model for receiving PORT (Table 2). The proportional contribution to the model was highest for the pT3-pT4 classification (OR 23.3) compared to the other predictor variables (Fig. 2b). Other variables that were significantly associated with receiving PORT were subsite gum compared to tongue (OR 2.58), subsite tongue compared to the other category (OR 2.86), margin 1–5 mm (OR 3.72), WPOI 4–5 (OR 1.83), and perineural invasion (OR 4.75). Patients were significantly less likely to receive local PORT per year increase of age (OR 0.96) and in case of vaso-invasive growth (OR 0.19).

**Table 2:** Multivariable (imputed) regression analysis for receiving postoperative radiotherapy.

Characteristic	OR	95% CI	p-value^a^
Sex			
Male	—	—	
Female	0.81	0.49, 1.34	0.42
Age in years	0.96	0.94, 0.98	**<0.001**
WHO score			
0–1	—	—	
2–4	0.50	0.19, 1.30	0.15
ASA score			
I-II	—	—	
III-V	1.65	0.93, 2.91	0.084
Subsite oral cavity			
Tongue	—	—	
Gum	2.58	1.29, 5.15	**0.007**
Floor of mouth	1.93	0.95, 3.91	0.069
Other subsites	2.86	1.40, 5.82	**0.004**
pT-classification			
pT1-T2	—	—	
pT3-pT4	23.3	13.1, 41.4	**<0.001**
Definitive surgical margin			
Margin > 5 mm	—	—	
Margin 1–5 mm	3.72	2.04, 6.78	**<0.001**
WPOI 4–5			
Not present	—	—	
Present	1.83	1.10, 3.05	**0.020**
Perineural invasion			
Not present	—	—	
Present	4.75	2.55, 8.87	**<0.001**
Vaso-invasive growth			
Not present	—	—	
Present	0.19	0.04, 0.88	**0.033**

Abbreviations: ASA = American Society of Anesthesiologists, OR = odds ratio, WHO = World Health Organization

a Significant p-values are printed in bold.

### Hospital variation in PORT treatment

**Fig. 3:**
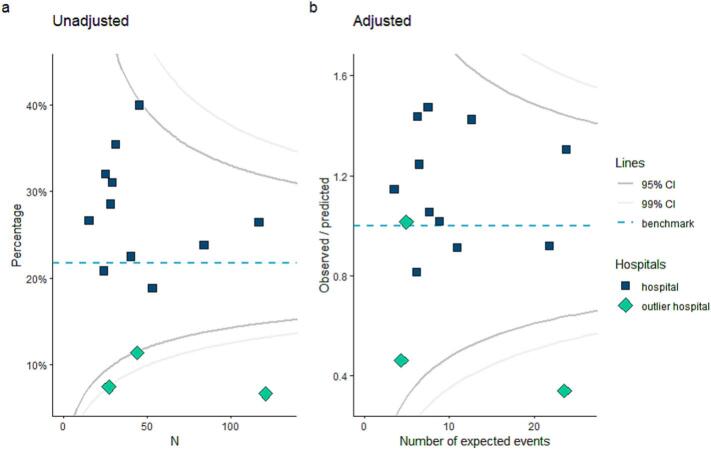
Funnel plots for hospital variation in the percentage of patients with adverse pathological features that received postoperative radiotherapy before (a) and after (b) correction for patient and tumor characteristics*. *Included patient and tumor variables were sex, age, World Health Organization status, ASA score, pT-classification, subsite oral cavity, margin status, WPOI 4–5, perineural invasion, vaso-invasive growth.

The percentage of patients receiving PORT varied from 6.6% to 40% on hospital-level (Fig. 3). Three centers were marked as outlier hospital because these treated significantly less patients with PORT compared to the benchmark (95%CI). After correction for patient and tumor characteristics, the results of one outlier remained outside the confidence intervals.

### PORT and survival outcomes

**Fig. 4:**
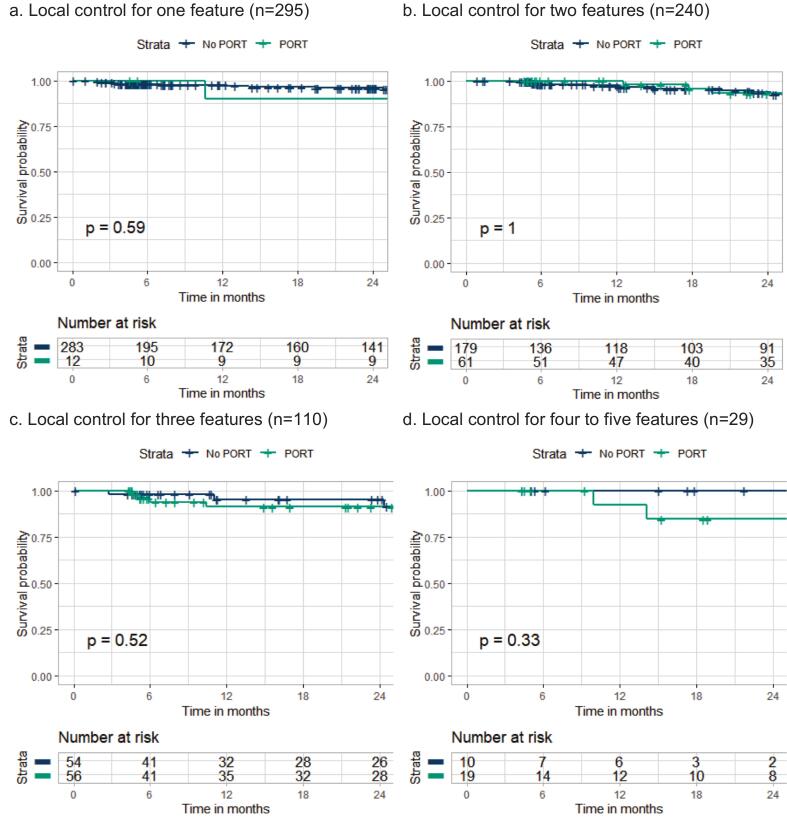
Kaplan Meier curves for two year local control per number of intermediate-risk adverse pathological features stratified for receiving PORT.* *Patients who had missing follow-up data (n = 9) or had the event of interest within six weeks after surgery (n = 5) were excluded (2.0%).

The Kaplan Meier curves per number of intermediate-risk adverse pathological features did not significantly differ for patients who received PORT regarding two-year LC (Fig. 4). The aHRs for receiving PORT were 1.56 for LC (95%CI 0.49–4.14, p = 0.36) and 0.55 for OS (95%CI 0.27–1.09, p = 0.084). Full univariable and multivariable Cox regression outcomes are described in Appendix C and D respectively.

## Discussion

This national cohort study evaluated the treatment with postoperative radiotherapy (PORT) in 683 first primary oral squamous cell carcinoma (OSCC) patients with intermediate-risk adverse pathological tumor features. These features are not assigned a relative importance in current international guidelines. In our study, PORT was given to patients with pT3-pT4 classification. Significant differences existed for PORT treatment between hospitals. There was no significant improvement in two-year local control and overall survival after PORT.

The NCCN and ESMO recommendations for PORT indication are based on retrospective cohort studies as randomized trials are lacking.[1], [3] However, many of these retrospective studies are based on small single center cohorts, lack correction for confounder variables, and report conflicting results.[18], [19], [20], [21], [22], [23] Three reviews on the survival outcomes after PORT have been performed. For pT1-2 N0-1 OSCC, 15 studies on PORT were included by *Ivaldi et al*. but no conclusions could be drawn due to the quality of the data.[6] *Best et al*. described five retrospective studies on stage I-II buccal OSCC and found no survival benefit after PORT.[5] The systematic review by *Liu et al.* concluded that PORT indication in pT1-4 OSCC could not be supported based on the presence of perineural or lympho-vascular invasion or unfavorable grade. Moreover, the authors expressed caution regarding overtreatment and unnecessary radiotherapy toxicity.[4] Upon first glance, the use of PORT in our cohort seems relatively low. When comparing this cohort to international data, it is important to realize that all patients with a clear indication for PORT have been excluded, such as surgical margins < 1 mm and pN2a or higher. In the Netherlands, it is common practice to indicate PORT when multiple intermediate risk factors are present. Therefore, the low use of PORT in the group of patients with only one or two features was expected. When more features were present, the majority of patients did receive PORT.

The association between patient and tumor characteristics and PORT treatment in our study aligned with previous literature. The most contributing factor was pT3-pT4 classification because many hospitals tend to indicate PORT when this is the only adverse pathological feature. Other hospitals only treat patients with PORT when multiple features are present. In the multivariable Cox regression analysis, two-year outcomes did not differ between pT1-pT2 and pT3-pT4 group (Appendix D). Another study on the same cohort indicated that pT3-pT4 tumors were more often associated with margins of < 1 and 1–5 mm. Despite correcting for this in the multivariable regression, pT3-pT4 classification contributed most.[24] The clinical relevance of margins between 1–5 mm remains a topic of discussion and studies have shown that 3 mm can be accepted as well.[19], [25] WPOI 4-5 and *peri*-neural growth are associated with local disease recurrence and are therefore considered as an indication for PORT. Vaso-invasive growth was associated with less PORT treatment in our cohort, which presumably could be because this factor is associated with nodal and distant metastasis instead of local failure.[3] Our results suggest that tongue tumors are less often treated by local PORT, which aligns with previous research on survival differences between the oral cavity subsites.[26] Contributing to this difference may be that radical resections are more often achieved in tongue tumors.[24] However, results were corrected for definitive margin status in the multivariable regression. There was a group of patients with multiple adverse pathological features that did not receive PORT. Because this study used registry data the specific reasoning for omitting PORT could not be retrieved. Older age was, as expected, associated with lower PORT use. In older patients, other health priorities often influence treatment decisions, particularly given the intensity of head and neck surgery.[27] Many patients opt for a “wait and see” instead of radiotherapy after counselling on the benefits of local PORT. The WHO score did not significantly contribute to less PORT treatment, however previous studies did indicate comorbidity and patient status influence therapeutic decisions.[28].

Due to ethical considerations, the reported outcomes of interest have not been investigated in a randomized clinical trial. We therefore performed a retrospective evaluation of real world data.[29] Following this, some bias need to be considered. First, there is the possibility of immortal time bias, which was considered of less influence as only five patients in our cohort died or had local recurrence/residual of disease < 6 weeks after surgery. Because patients are registered as-treated in the DHNA, indication bias could be of influence. However, we were able to correct for multiple confounders in the regression analyses. Pathological inter-observer variability could be of influence, especially because data from multiple hospitals was combined in our study.[30] Moreover, the DHNA data does not distinguish between local or locoregional PORT. It must be noted that only administered PORT is registered in the DHNA database, not the advice for PORT given to the patient, based on the postoperative multidisciplinary discussion. We assume that the refusion of patients to receive PORT was evenly distributed over all hospitals. We only included patients with an indication for PORT based on local tumor variables, as patients with adverse pathological features regarding neck metastases were excluded. Regional and distant control were not included as study outcomes because the main focus was on local PORT. An additional analyses demonstrated no significant improvement in two-year disease free survival after PORT (Appendix E). Local hospital protocols differ on the treatment of only local or locoregional PORT for indications related to features of the primary tumor. A recent study indicated that for pN0 patients, regional control was comparable with and without of elective neck radiotherapy.[31] We therefore do not think that the inability to distinguish between local and locoregional PORT has influenced the study outcomes. The DHNA database currently only contains two-year follow-up data for oral cavity carcinoma; the five-year data will be included in the future. Longer follow-up data is needed to further elucidate the long term effect of PORT for our study population.

Our study underlines the difficulty in adequate patient selection for PORT after oral cancer surgery. The cohort that received PORT exhibited a higher prevalence of intermediate-risk adverse pathological features, and PORT was most frequently administered in cases involving pT3–pT4 tumors. Taking this selection bias into account, the observed absence of a significant effect of PORT on local control and survival can be interpreted in two ways. First, one might argue that PORT enabled patients with a greater burden of risk factors to achieve outcomes comparable to those who did not receive PORT. However, the multivariable Cox regression analysis adjusted for all individual intermediate-risk pathological features. Therefore, an alternative interpretation is that patients who received PORT may have achieved similar outcomes even without its administration. Both interpretations underscore the need for more precise patient selection, especially because PORT is associated with considerable toxicity. Moreover, as healthcare expenses rise and patient loads for radiotherapy departments increase, resources should be allocated to patients who benefit the most. Future studies should therefore focus on consensus discussions for guideline adherence and treatment allocation of PORT in OSCC. A prospective cohort study comparing treatment outcomes between hospitals or countries with differing PORT treatment guidelines could clarify the importance of the adverse criteria without randomizing patients. However, large patient samples would be needed, data gathering would be time-intensive, and bias remains present. Still, real-world data can provide valuable insights in situations where randomized trials are either unethical or undesirable.

## Conclusions

PORT treatment in OSCC patients with intermediate-risk adverse pathological tumor features is executed mostly in pT3-pT4 tumors. Our study highlighted between-hospital differences in criterium adherence for PORT indication after correction for patient and tumor characteristics. Significant improvement in two-year local control and overall survival with PORT could not be confirmed. Future research should focus on guideline consensus for treatment allocation to prevent over- or undertreatment.

## Research ethics and patient consent

This research proposal was reviewed by the Institutional research review board Erasmus Medical Center (Rotterdam, The Netherlands), and the board confirmed that the rules laid down in the Medical Research Involving Human Subjects Act do not apply to this research proposal (MEC-2022–0816).

## CRediT authorship contribution statement

**Hanneke Doremiek van Oorschot:** Conceptualization, Data curation, Formal analysis, Investigation, Methodology, Project administration, Validation, Visualization, Writing – original draft, Writing – review & editing. **Jose Angelito Hardillo:** Conceptualization, Data curation, Formal analysis, Methodology, Supervision, Writing – original draft, Writing – review & editing. **Frank J.P. Hoebers:** Conceptualization, Formal analysis, Methodology, Supervision, Writing – original draft, Writing – review & editing. **Joris B.W. Elbers:** Formal analysis, Methodology, Writing – original draft, Writing – review & editing. **Robert Jan Baatenburg de Jong:** Conceptualization, Data curation, Methodology, Supervision, Writing – original draft, Writing – review & editing. **Robert J.J. van Es:** . **Guido B. van den Broek:** . **Robert Paul Takes:** . **Gyorgy Bela Halmos:** . **Dominique Valerie Clarence de Jel:** . **Richard Dirven:** . **Martin Lacko:** . **Lauretta Anna Alexandra Vaassen:** . **Jan-Jaap Hendrickx:** . **Marjolijn Abigal Eva-Maria Oomens:** . **Hossein Ghaeminia:** . **Jeroen C. Jansen:** . **Annemarie Vesseur:** . **Rolf Bun:** . **Leonora Q. Schwandt:** . **Christiaan A. Krabbe:** . **Thomas J.W. Klein Nulent:** . **Alexander Jan Marcelis van Bemmel:** . **Reinoud J. Klijn:** .

## Funding

This manuscript did not receive a grant from any funding agency in the public, commercial or not-for-profit sectors.

## Declaration of Competing Interest

The authors declare that they have no known competing financial interests or personal relationships that could have appeared to influence the work reported in this paper.
