# Supplementary material for: Indication adherence and outcome of post-operative radiotherapy in oral cavity cancer patients with intermediate adverse pathological tumor features: A nationwide population-based analysis
Source: Clin Transl Radiat Oncol. 2026 Apr 4;59:101157. doi: 10.1016/j.ctro.2026.101157 (PMC13091407; doi:10.1016/j.ctro.2026.101157)
Supplement: Supplementary Data 1 [file mmc1.docx]

**Appendix A**

Distribution of propensity scores in the cohort of patients with adverse pathological features (n=683). Treated units are patients who were treated with local postoperative radiotherapy.


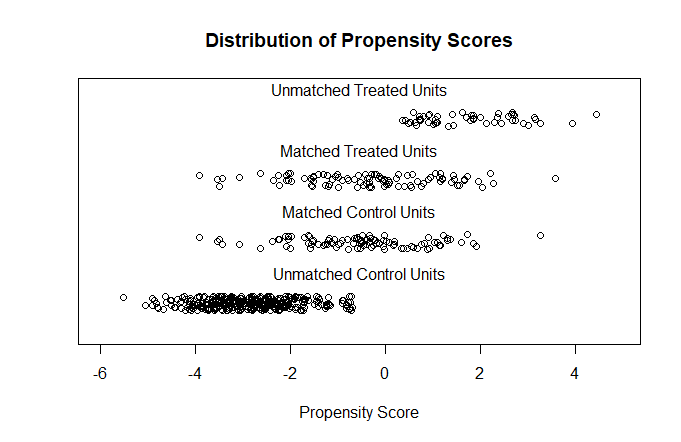


**Appendix B**

Flow chart for study inclusion


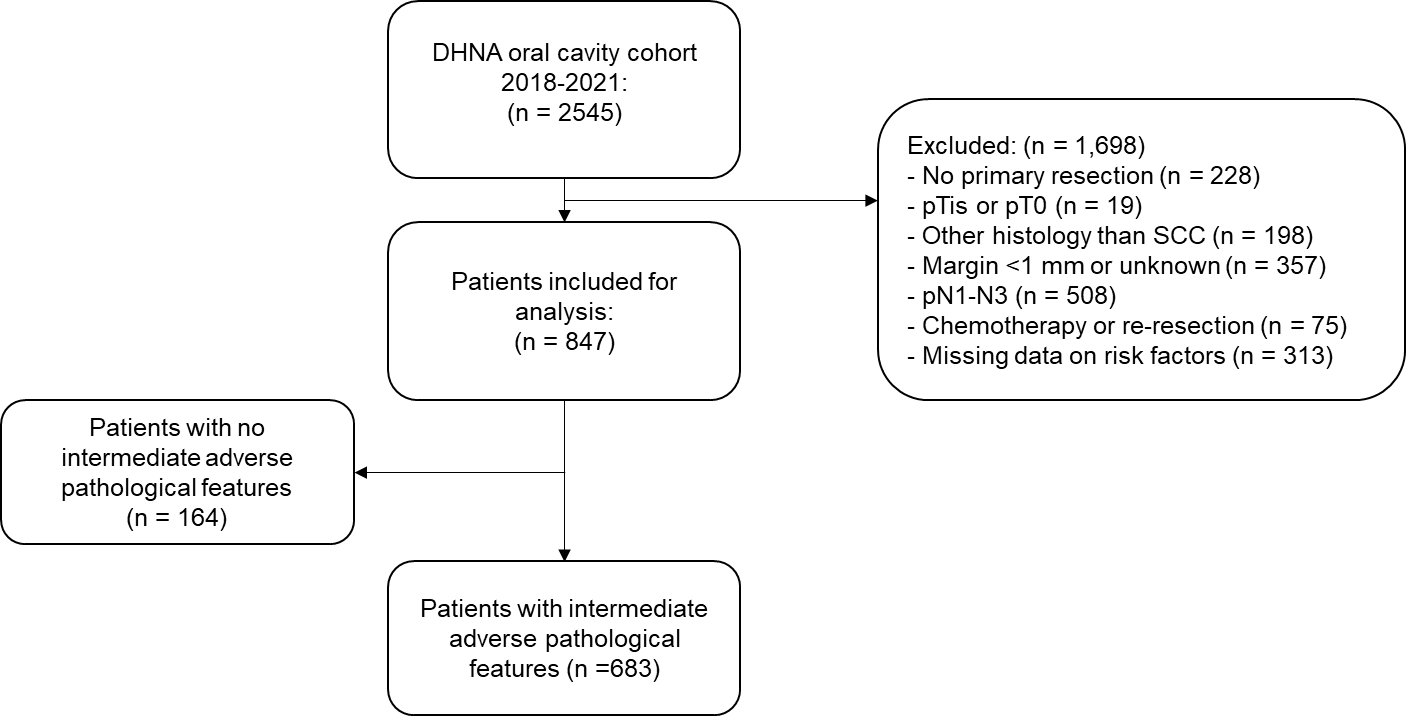


Abbreviations: DHNA = Dutch Head and Neck Audit, SCC = squamous cell carcinoma

**Appendix C**

Univariable (imputed) Cox regression analysis for two-year survival outcomes.

|  | 2y-Overall Survival | | | 2y-Local Control | | |
| --- | --- | --- | --- | --- | --- | --- |
| Characteristic | HR*^1^* | 95% CI*^1^* | p-value | HR*^1^* | 95% CI*^1^* | p-value |
| PORT |  |  |  |  |  |  |
| No | — | — |  | — | — |  |
| Yes | 0.65 | 0.37, 1.13 | 0.12 | 1.57 | 0.77, 3.19 | 0.21 |
| Sex |  |  |  |  |  |  |
| Male | — | — |  | — | — |  |
| Female | 1.02 | 0.68, 1.55 | 0.91 | 1.36 | 0.70, 2.65 | 0.35 |
| Age in years | 1.06 | 1.04, 1.08 | **<0.001** | 1.03 | 1.00, 1.06 | 0.062 |
| WHO score |  |  |  |  |  |  |
| 0-1 | — | — |  | — | — |  |
| 2-4 | 3.91 | 2.41, 6.33 | **<0.001** | 0.76 | 0.17, 3.35 | 0.70 |
| ASA score |  |  |  |  |  |  |
| I-II | — | — |  | — | — |  |
| III-V | 2.33 | 1.51, 3.59 | **<0.001** | 1.12 | 0.56, 2.22 | 0.75 |
| pT-classification |  |  |  |  |  |  |
| pT1-T2 | — | — |  | — | — |  |
| pT3-pT4 | 1.24 | 0.80, 1.93 | 0.33 | 1.02 | 0.50, 2.12 | 0.95 |
| Subsite oral cavity |  |  |  |  |  |  |
| Tongue | — | — |  | — | — |  |
| Gum | 1.85 | 1.08, 3.19 | **0.026** | 2.26 | **1.01, 5.02** | **0.046** |
| Floor of mouth | 1.10 | 0.62, 1.95 | 0.74 | 0.76 | 0.27, 2.15 | 0.59 |
| Other subsites | 1.26 | 0.69, 2.28 | 0.44 | 1.18 | 0.45, 3.12 | 0.73 |
| Definitive surgical margin |  |  |  |  |  |  |
| Margin >5mm | — | — |  | — | — |  |
| Margin 1-5mm | 0.92 | 0.58, 1.45 | 0.72 | 0.96 | 0.47, 1.99 | 0.91 |
| WPOI 4-5 |  |  |  |  |  |  |
| Not present | — | — |  | — | — |  |
| Present | 1.10 | 0.71, 1.68 | 0.67 | 1.46 | 0.72, 2.97 | 0.28 |
| Perineural invasion |  |  |  |  |  |  |
| Not present | — | — |  | — | — |  |
| Present | 1.55 | 0.95, 2.55 | 0.080 | **2.11** | **1.02, 4.36** | **0.044** |
| Vaso-invasive growth |  |  |  |  |  |  |
| Not present | — | — |  | — | — |  |
| Present | 1.49 | 0.68, 3.25 | 0.31 | 0.46 | 0.06, 3.63 | 0.45 |

*Patients who had missing follow-up data or had the event of interest within six weeks after surgery were excluded (2.0%, n=14).

Abbreviations: PORT = post-operative radiotherapy, WPOI = worst pattern of invasion

**Appendix D**

Multivariable (imputed) Cox regression analysis for two-year survival outcomes.

|  | Overall survival | | | Local control | | |
| --- | --- | --- | --- | --- | --- | --- |
| Characteristic | HR*^1^* | 95% CI*^1^* | p-value | HR*^1^* | 95% CI*^1^* | p-value |
| PORT |  |  |  |  |  |  |
| No | — | — |  | — | — |  |
| Yes | 0.55 | 0.27, 1.09 | 0.084 | 1.56 | 0.59, 4.14 | 0.36 |
| Sex |  |  |  |  |  |  |
| Male | — | — |  | — | — |  |
| Female | 0.74 | 0.48, 1.15 | 0.18 | 1.24 | 0.62, 2.48 | 0.53 |
| Age in years | 1.05 | 1.03, 1.07 | **<0.001** | 1.03 | 0.99, 1.06 | 0.12 |
| WHO score |  |  |  |  |  |  |
| 0-1 | — | — |  | — | — |  |
| 2-4 | 2.35 | 1.26, 4.39 | **0.008** | 0.63 | 0.12, 3.34 | 0.56 |
| ASA score |  |  |  |  |  |  |
| I-II | — | — |  | — | — |  |
| III-V | 1.28 | 0.75, 2.20 | 0.36 | 0.92 | 0.42, 2.02 | 0.84 |
| pT-classification |  |  |  |  |  |  |
| pT1-T2 | — | — |  | — | — |  |
| pT3-pT4 | 1.47 | 0.83, 2.60 | 0.18 | 0.53 | 0.20, 1.46 | 0.21 |
| Subsite oral cavity |  |  |  |  |  |  |
| Tongue | — | — |  | — | — |  |
| Gum | 1.65 | 0.86, 3.17 | 0.13 | 2.66 | 0.98, 7.20 | 0.055 |
| Floor of mouth | 1.26 | 0.69, 2.30 | 0.44 | 0.79 | 0.26, 2.33 | 0.65 |
| Other subsites | 1.28 | 0.68, 2.41 | 0.43 | 1.23 | 0.44, 3.49 | 0.68 |
| Definitive surgical margin |  |  |  |  |  |  |
| Margin >5mm | — | — |  | — | — |  |
| Margin 1-5mm | 1.10 | 0.67, 1.82 | 0.70 | 0.90 | 0.40, 2.03 | 0.79 |
| WPOI 4-5 |  |  |  |  |  |  |
| Not present | — | — |  | — | — |  |
| Present | 1.05 | 0.66, 1.68 | 0.82 | 1.39 | 0.63, 3.05 | 0.40 |
| Perineural invasion |  |  |  |  |  |  |
| Not present | — | — |  | — | — |  |
| Present | 1.72 | 1.00, 2.95 | 0.051 | 2.14 | 0.95, 4.81 | 0.065 |
| Vaso-invasive growth |  |  |  |  |  |  |
| Not present | — | — |  | — | — |  |
| Present | 1.21 | 0.52, 2.80 | 0.65 | 0.42 | 0.05, 3.49 | 0.41 |

*Patients who had missing follow-up data or had the event of interest within six weeks after surgery were excluded (2.0%, n=14).

Abbreviations: PORT = post-operative radiotherapy, WPOI = worst pattern of invasion

**Appendix E**

Multivariable (imputed) Cox regression analysis for two-year disease free survival outcomes.

|  | Disease free survival | | |
| --- | --- | --- | --- |
| Characteristic | HR*^1^* | 95% CI*^1^* | p-value |
| PORT |  |  |  |
| No | — | — |  |
| Yes | 0.67 | 0.37, 1.23 | 0.19 |
| Sex |  |  |  |
| Male | — | — |  |
| Female | 0.78 | 0.53, 1.16 | 0.22 |
| Age in years | 1.04 | 1.03, 1.06 | <0.001 |
| WHO score |  |  |  |
| 0-1 | — | — |  |
| 2-4 | 0.81 | 0.41, 1.59 | 0.54 |
| ASA score |  |  |  |
| I-II | — | — |  |
| III-V | 1.12 | 0.73, 1.72 | 0.61 |
| pT-classification |  |  |  |
| pT1-T2 | — | — |  |
| pT3-pT4 | 1.02 | 0.60, 1.74 | 0.94 |
| Subsite oral cavity |  |  |  |
| Tongue | — | — |  |
| Gum | 1.45 | 0.82, 2.53 | 0.2 |
| Floor of mouth | 0.69 | 0.38, 1.25 | 0.22 |
| Other subsites | 1.16 | 0.67, 2.01 | 0.6 |
| Definitive surgical margin |  |  |  |
| Margin >5mm | — | — |  |
| Margin 1-5mm | 1.04 | 0.66, 1.65 | 0.86 |
| WPOI 4-5 |  |  |  |
| Not present | — | — |  |
| Present | 0.98 | 0.64, 1.48 | 0.91 |
| Perineural invasion |  |  |  |
| Not present | — | — |  |
| Present | 1.44 | 0.87, 2.38 | 0.15 |
| Vaso-invasive growth |  |  |  |
| Not present | — | — |  |
| Present | 0.65 | 0.23, 1.81 | 0.41 |

*Patients who had missing follow-up data or had the event of interest within six weeks after surgery were excluded (2.0%, n=14).

Abbreviations: PORT = post-operative radiotherapy, WPOI = worst pattern of invasion
